# Is the lymph node ratio superior to the Union for International Cancer Control (UICC) TNM system in prognosis of colon cancer?

**DOI:** 10.1186/1477-7819-11-79

**Published:** 2013-03-23

**Authors:** Leif Schiffmann, Anne Karen Eiken, Michael Gock, Ernst Klar

**Affiliations:** 1 Department of General, Thoracic, Vascular and Transplantation Surgery, University of Rostock, Schillingallee 35, Rostock 18057, Germany

**Keywords:** Colon cancer, TNM system, ln ratio

## Abstract

**Background:**

Decision making for adjuvant chemotherapy in stage III colon cancer is based on the TNM system. It is well known that prognosis worsens with higher pN classification, and several recent studies propose superiority of the lymph node ratio (ln ratio) to the TNM system. Therefore, we compared the prognosis of ln ratio to TNM system in our stage III colon cancer patients.

**Methods:**

A total of 939 patients underwent radical surgery for colorectal cancer between January 2000 and December 2009. From this pool of patients, 142 colon cancer stage III patients were identified and taken for this analysis. Using martingale residuals, this cohort could be separated into a group with a low ln ratio and one with a high ln ratio. These groups were compared to pN1 and pN2 of the TNM system.

**Results:**

For ln ratio, the cutoff was calculated at 0.2. There was a good prognosis of disease-free and cancer-related survival for the N-category of the TNM system as well as for the lymph node ratio. There was no statistical difference between using the N-category of the TNM system and the ln ratio.

**Conclusions:**

There might not be a benefit in using the lymph node ratio rather than the N category of the TNM system as long as the number of subgroups is not increased. In our consideration, there is no need to change the N categorization of the TNM system to the ln ratio.

## Background

Colorectal cancer (CRC) is one of the most important causes of cancer-related death in the western world. In Germany, approximately 71,400 patients develop CRC per year [1]. Most cancer related deaths are not caused by the primary cancer site, but by distant metastasis. However, patients without distant metastasis at the time of surgery (UICC I to III) still have improvable 5-year survival rates between 41% and 96% [2,3].

Adjuvant or palliative (radio) chemotherapy is generally recommended for UICC III and IV tumors despite the associated toxicities [4,5]. Patients’ outcomes vary widely between stages with a worsen outcome from stage I to IV, but also within each stage. So far, indication for adjuvant therapy is based on the TNM system and therefore basically on the lymph node status. Several recent studies propose superiority of the lymph node ratio (ln ratio) to the TNM system in prognosis of colorectal cancer [6-11]. The ln ratio is the ratio of the number of positive nodes to the total number of nodes excised. In this study we compared retrospectively the prognosis of ln ratio to TNM system in our stage III colon cancer patients in terms of disease-free survival (DFS) and cancer-related survival.

## Methods

### Patients

From January 2000 to December 2009, 939 patients underwent radical surgery for CRC in the Department of General, Thoracic, Vascular und Transplantation Surgery. All patients were treated according to standard treatment guidelines [12]. A preoperative anesthesiological evaluation was obtained according to the American Society of Anesthesiologists (ASA) general classification [13] to determine state of health and comorbidity. Preoperative staging involved endoscopy and biopsy, abdomen ultrasound and chest radiography or computer tomography of abdomen, chest or both.

Patients were staged according to the TNM system [14]. Clinical data were retrieved retrospectively. Data recorded included gender, type of admission, comorbidities, ASA score, tumor characteristics, type of resection, morbidity and 30-day mortality. Follow-up information was recorded regarding recurrence and distant metastasis, overall survival and cancer-related survival as well as information about adjuvant therapy in early 2011. Follow-up examinations were carried out in cooperation with the referring physicians according to the German S3 guidelines for colorectal cancer [12] and provided a comprehensive and complete data collection.

From this pool of patients, all 142 patients with stage III colon cancer were identified and included in this study.

The study was approved by the Medical Ethical Committee of Rostock University.

#### Comparing lymph node ratio to N category of the TNM system and statistical analysis

To be able to compare the N category of the TNM system to the ln ratio, we decided to separate the ln ratio of all patients into two groups. Therefore, martingale residuals [15] were calculated and represented by a smoothed residual plot [16] to determine if and which cutoff value of the ln ratio would allow the best separation of the groups of patients with worse and better survival.

Statistical analysis was performed using Statistical Package for Social Science (SPSS™) version 15.0. (http://www.spss.com). Statistical analysis was done using Pearson’s chi-square test of Fisher’s exact test. Survival curves were calculated according to the Kaplan-Meier method. Survival curves were tested for significant differences using the log-rank test. A *P* value of <0.05 was considered as statistically significant. Additionally, further analysis was preformed for the N category of the TNM system and the ln ratio.

## Results

From 939 patients a cohort of 142 stage III colon cancer patients was identified. Patients and tumor characteristics are shown in Table 1. On average, there were 23.2 lymph nodes harvested and of these, 4 lymph nodes tested positive for metastasis. In the specimens of 13 patients, fewer than 12 lymph nodes were analyzed. Follow-up time was slightly less than 4 years (mean), and just about one-third of all patients developed recurrence of cancer; 30% of ALL patients died.

**Table 1:** Patients’ and tumor characteristics of the 142 patients included in the analysis

	**142 Patients (%)**	
**Mean age (years), (range)**	**70.0 (37 to 94)**	
Gender ratio (f/m)	1 : 1	(50:50)
Localization		
Cecum	19	(13.4)
Right hemicolon	37	(26.1)
Transverse colon	20	(14.1)
Left hemicolon	8	(5.6)
Sigmoid colon	58	(40.8)
Comorbidities	117	(82.4)
Emergency surgery	25	(17.6)
ASA Score		
1 to 2	71	(50.0)
3 to 4	71	(50.0)
Tumor characterization		
T1	3	(2.1)
T2	14	(9.9)
T3	64	(45.1)
T4	61	(43.0)
R1/2	10	(7.0)
N1	80	(56.3)
N2	62	(43.7)
Lymph nodes examined	23.16	± 9.33
Lymph nodes positive	4.04	± 4.15
V0	73	(51.4)
L0	76	(53.5)
G1/2	111	(78.2)
Adjuvant chemotherapy	126	(88.7)
Follow-up (years)	3.85	±2.81
Recurrence	48	(33.8)
Died of disease	43	(30.3)

*ASA*, American Society of Anesthesiologists.

Estimated cancer-related 5-year survival was 66.8% and estimated 5-year disease-free survival was 64.2%.

**Figure 1:**
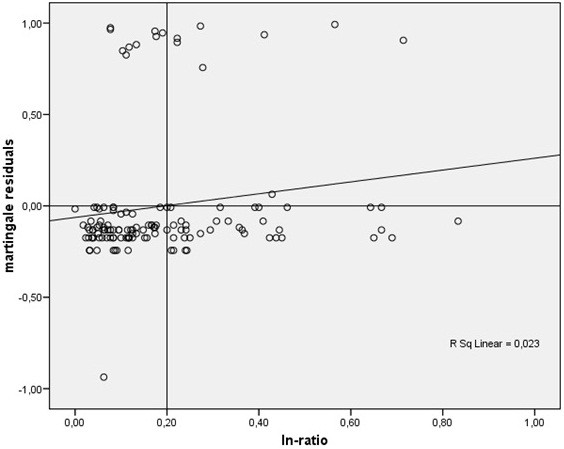
**Martingale residuals as a function of the lymph node ratio. **Each dot represents the difference between the observed individual status and the calculated cumulative risk at the end of the observation period.

To determine, if there was a potential benefit of calculating the lymph node ratio rather than using the N category of the TNM system, we separated the patient cohort into two groups of lymph node ratios using the martingale residuals analog to the two groups of the N category of the TNM system. The cutoff was at 0.2 positive to all examined lymph-nodes (shown in Figure 1).

**Figure 2:**
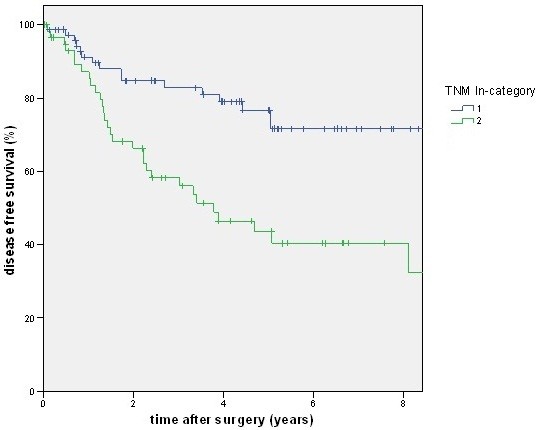
**The figure shows the disease-free survival stratified by pN category. **With a higher stage (pN2) disease-free survival (DFS) becomes worse (*P *<0.001).

**Figure 3:**
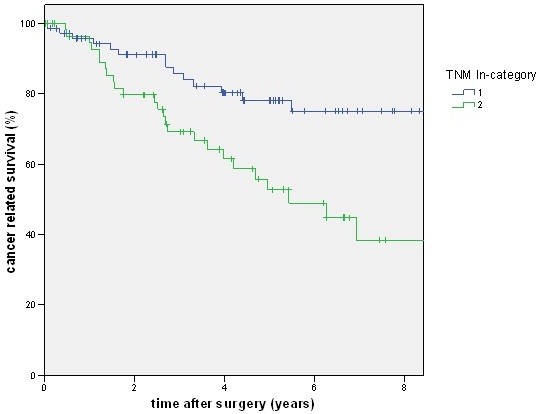
**The figure shows the cancer-related survival stratified by pN category. **With a higher stage (pN2) cancer-related survival becomes worse (*P *= 0.002).

**Figure 4:**
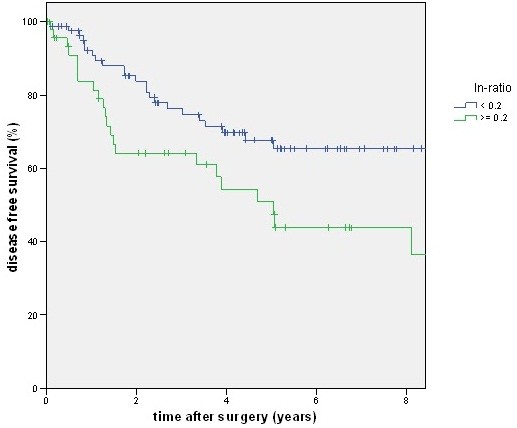
**The figure shows the disease-free survival stratified by lymph node ratio. **With a higher ratio disease-free survival (DFS) becomes worse (*P *= 0.008).

**Figure 5:**
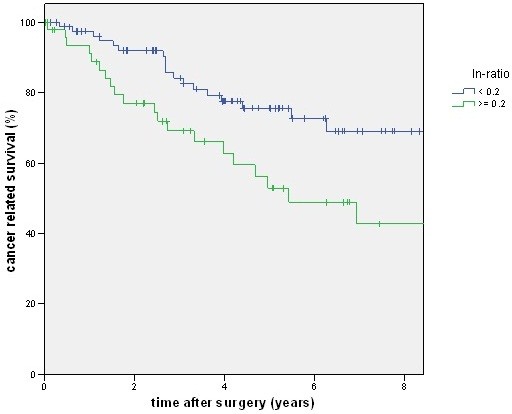
**The figure shows the cancer-related survival stratified by lymph node ratio. **With a higher ratio cancer-related survival becomes worse (*P *= 0.007).

A total of 88 patients had a lymph node ratio below 0.2, but only 80 patients had an N1 category. There was a good prognosis of disease-free and cancer-related survival for the N category of the TNM system (Figures 2 and 3) as well as for the lymph node ratio (Figures 4 and 5). But as shown in the figures and posted in Table 2, statistically, differentiation was stronger using the N category of the TNM system.

**Table 2:** Comparison of lymph node ratio (LNR) and pN category of the TNM system for disease-free survival (DFS) and cancer-related survival (CRS)

		**DFS (%)**	**Mean DFS (years)**^**a**^	95% CI*	***P***
LNR	<0.20	73.9	7.54	6.60 to 8.48	0.008
	≥0.20	53.8	5.35	4.05 to 6.65	
N	1	78.8	8.07	7.13 to 9.02	< 0.001
	2	50.0	4.85	3.82 to 5.87	
		**CRS (%)**	Mean CRS (years)^*^	95% CI*	***P***
LNR	<0.20	78.4	7.92	7.01 to 8.84	0.007
	≥0.20	55.8	5.85	4.61 to 7.09	
N	1	78.8	8.18	7.23 to 9.13	0.002
	2	58.1	5.41	4.53 to 6.29	

^a^Estimation is limited to longest follow-up time.

There is no statistical difference between pN category of the TNM system and the ln ratio, although the differences in our cohort are bigger for the pN category of the TNM system, as is shown by a lower *P* value. It is noteworthy that 12 patients with pN1 were categorized in the high ln ratio group, and 20 patients with pN2 were categorized in the low ln ratio group.

## Discussion

We retrospectively analyzed whether it would be beneficial to determine the lymph node ratio rather than the N category of the TNM system in prognosis of colon cancer. Therefore, we picked 142 stage III colon cancer patients from a cohort of 939 colorectal cancer patients who were operated on over a 10-year period. In comparison to other reports [6], the fraction, Stage III patients in comparison to all (I to IV) patients) is rather small but the number of examined lymph nodes is rather high [8,10] as is the percentage of patients receiving adjuvant chemotherapy [17].

For easier comparison of N stages to lymph node ratio, we decided to calculate two groups of lymph node ratios. By doing so, there is no superiority in predicting disease-free and overall survival of the lymph node ratio to the N category of the TNM system. Other authors split patient cohorts by random or percentiles rather than calculating groups [6,7,9,11]. By using more groups, the lymph node ratio gains in precision of prognosis [6,7,11], but if more subgroups were established in the N category, precision would presumably rise there as well. So, in our opinion, the only possibility for comparing the two approaches is to use the same number of groups.

To exclude the bias of neoadjuvant treated patients, we decided not to include rectal cancer patients in the analysis. In rectal cancer patients, the number of pathologically diagnosed lymph nodes is frequently lowered after neoadjuvant treatment in comparison to patients not treated with neoadjuvants [18]. Therefore, all rectal cancer patients were excluded from this analysis. Other authors used a stage I to IV colorectal cancer cohort over many years to show the advantage of the lymph node ratio [11]. The disadvantage of this particular approach is that there are many patients included who do not have lymph node metastasis at all, have distant metastasis synchronously and that patients were operated over a long time period. Within this period, surgical techniques might have changed substantially, and therefore, it might be difficult to compare patients’ courses of the disease.

## Conclusions

Conclusively, we show with a rather simple approach, that using the lymph node ratio rather than the N category of the TNM system is not beneficial in terms of predicting overall and disease-free survival. Of course there will be an advantage by increasing the number of subgroups within the category - in the N category of the TNM system as well as within the lymph node ratio. In our consideration, there is no need to change the categorization toward the lymph node ratio.

## Abbreviations

ASA: American Society of Anesthesiologists; CRC: Colorectal cancer; CRS: Cancer-related survival; DFS: Disease-free survival; ln ratio: Lymph node ratio; UICC: Union for International Cancer Control.

## Competing interests

The authors declare that they have no conflicts of interest.

## Authors’ contributions

LS and AKE conceived and coordinated the study, collected patients’ data and participated in the statistical analysis. LS drafted the manuscript. MG and EK participated in preparing and drafting the manuscript. All authors read and approved the final manuscript.
